# Nutrients, Phytochemicals, and In Vitro Antioxidant and Antimicrobial Activities of Lulo (*Solanum quitoense* Lam.) Fruit Pulp, Peel, and Seeds

**DOI:** 10.3390/foods14122083

**Published:** 2025-06-13

**Authors:** Mikel Añibarro-Ortega, Maria Inês Dias, Jovana Petrović, Alexis Pereira, Marina Soković, Lillian Barros, José Pinela

**Affiliations:** 1 CIMO, LA SusTEC, Instituto Politécnico de Bragança, Campus de Santa Apolónia, 5300-253 Bragança, Portugal; 2 Nutrition and Bromatology Group, Department of Analytical and Food Chemistry, Faculty of Food Science and Technology, University of Vigo, Ourense Campus, E-32004 Ourense, Spain; 3 Institute for Biological Research “Siniša Stanković”, National Institute of Republic of Serbia, University of Belgrade, Bulevar despota Stefana 142, 11108 Belgrade, Serbia; 4 National Institute for Agricultural and Veterinary Research (INIAV), I.P., Rua dos Lágidos, Lugar da Madalena, Vairão, 4485-655 Vila do Conde, Portugal

**Keywords:** naranjilla/lulo, nutritional composition, phenolic compounds, phenolamides, antioxidant activity, functional food

## Abstract

Lulo or naranjilla (*Solanum quitoense* Lam.) is an Andean fruit with a sour and refreshing flavor, widely used in the preparation of juices and sweets. Despite its potential for international markets, it remains largely unknown outside its native regions, and most existing studies have focused on the whole fruit or its juice. This study investigated the nutritional and phenolic profiles of the peel, pulp, and seeds of *S. quitoense* using official food analysis methods and chromatographic techniques. In addition, the in vitro antioxidant activity and antimicrobial effects against foodborne fungi and bacteria were assessed. The peel was rich in ascorbic acid (25.2 mg/100 g fw), α-tocopherol (7.9 mg/100 g fw), dietary fiber (16.5 g/100 g fw), macrominerals (Na, Ca, K), and flavonoids (14.2 mg/g extract); the pulp contained high levels of citric acid (4.22 g/100 g fw) and sucrose (2.7 g/100 g fw); and the seeds stood out for their contents of trace elements (Zn, Cu, Mn, Fe), oleic acid, and spermidine-derived phenolamides (37.8 mg/g extract). Hydroethanolic extracts showed antioxidant activity by inhibiting lipid peroxidation and oxidative hemolysis, with the seed extract exhibiting the strongest antifungal effect against *Aspergillus versicolor*, likely due to its high spermidine derivative content. These findings shed light on the potential of *S. quitoense* fruit for the development of functional foods, antioxidant-rich beverages, and nutraceutical products.

## 1. Introduction

The global demand for tropical fruits with high nutritional value and distinctive organoleptic characteristics has surged in recent years, driven by their potential health benefits and increasing consumer interest in novel and functional foods. Among these fruits, *Solanum quitoense* Lam., commonly known as lulo or naranjilla, has garnered growing attention due to its unique acidic flavor, rich micronutrient profile, and abundance of bioactive phytochemicals [1]. Native to the Andean regions of South America, particularly Colombia, Peru, and Ecuador, *S. quitoense* is traditionally consumed fresh or processed into juices, cocktails, jams, and desserts, among other products. Today, its potential in innovative food applications is being explored [2,3].

Although still relatively unknown in Europe, *S. quitoense* fruit is a valuable source of vitamin C and essential minerals, including potassium, calcium, and magnesium. Additionally, it contains a diverse array of bioactive compounds, such as carotenoids and phenolic compounds, which contribute to its antioxidant capacity and health-promoting properties [4,5]. Of particular interest is its high polyphenol content, particularly phenolic acids, flavonoids, and phenolamides (spermidine derivatives), which have been associated with antioxidant, antimicrobial, and anti-inflammatory effects [6,7,8]. While scientific attention to *S. quitoense* is growing, most studies to date have focused on the whole fruit or its juice, with limited differentiation among its anatomical components, the pulp, peel, and seeds. This presents a research gap, as each fruit part may harbor a unique nutritional and phytochemical profile, influencing its functional properties and industrial applications.

Phenolamides (hydroxycinnamic acid amides, HCAA) stand out among the bioactive constituents of *S. quitoense* due to their strong antioxidant and antimicrobial activities [6,8]. These specialized metabolites are synthesized through the conjugation of hydroxycinnamic acids (e.g., caffeic acid) with polyamines such as spermidine. Beyond their role in plant defense, phenolamides have been connected with various health benefits, including neuroprotective and anti-inflammatory effects [7,8,9,10]. Although spermidine derivatives have previously been identified in *S. quitoense* [11], a detailed analysis of their specific distribution across the fruit’s anatomical parts remains unexplored. Additionally, this fruit contains significant levels of organic acids such as citric and malic acids, which not only contribute to the fruit’s characteristic acidity but also enhance its antioxidant properties [11,12]. Given the increasing interest in *S. quitoense* fruit as a functional food and a potential ingredient in nutraceutical and food supplements, a deeper understanding of the distribution and bioactivity of its key micronutrients and phytochemicals is needed.

Despite growing scientific focus on *S. quitoense*, a knowledge gap remains regarding the distinct nutritional and phytochemical composition of its different fruit parts. Previous studies have primarily examined the whole fruit or pulp [1], overlooking the unique bioactive compound profiles of the peel and seeds. To address this gap, the present study aimed to systematically characterize the nutritional composition, phenolic content, and in vitro bioactive properties of *S. quitoense* fruit pulp, peel, and seeds. Understanding these variations is crucial for optimizing the industrial applications of *S. quitoense*, promoting the valorization of its by-products (peel and seeds), and expanding its functional uses in innovative food and nutraceutical formulations. By providing a comprehensive evaluation of each fruit component, this study supports the sustainable utilization of *S. quitoense* fruit and reinforces its growing recognition as a functional food with significant health-promoting potential.

## 2. Materials and Methods

### 2.1. Chemicals, Standards, and Biological Materials

Table S1 provides a list of the chemicals, standards, and biological materials utilized in this study, while Table S2 outlines the equipment used, including the chromatographic instruments, their specifications, and the respective manufacturers.

### 2.2. Plant Material

**Figure 1:**
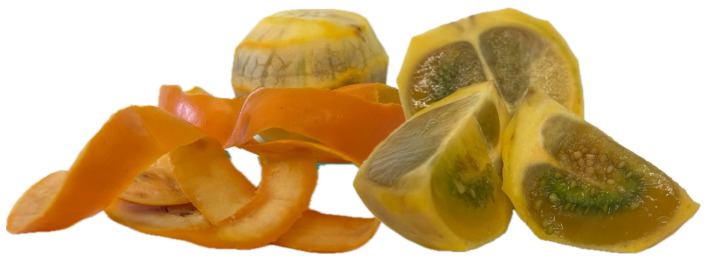
Peeled *Solanum quitoense* fruit.

Fresh *Solanum quitoense* Lam. fruits imported from Colombia were purchased in May 2022 from a local fruit market in Segovia, Spain. The fruits were carefully separated into peel (exocarp), pulp (mesocarp, endocarp, placenta, and locular gel), and seeds (Figure 1). Each fruit part was individually weighed to determine its relative contribution to the total fruit fresh weight (fw), with the pulp, peel, and seeds accounting for 75.8%, 12.4%, and 11.8% (*w*/*w*), respectively. The moisture content of each sample was determined using a moisture analyzer. The plant material was then freeze-dried to a constant weight, finely ground to ~20 mesh particle size, vacuum sealed, and stored at −20 °C until further analysis. Three independent samples were processed and analyzed separately.

### 2.3. Compositional Analysis

#### 2.3.1. Centesimal Composition and Energy

Protein, crude fat, ash, and total dietary fiber contents (g/100 g fresh weight (fw)) in the pulp, peel, and seed samples were determined following AOAC official methods [13]. Crude protein was estimated using the macro-Kjeldahl method (AOAC 920.152) and 6.25 as the nitrogen conversion factor. Crude fat was quantified gravimetrically after Soxhlet extraction with petroleum ether (AOAC 920.85). Ash content was determined by weight difference after incineration at 550 ± 15 °C (AOAC 940.26). Total dietary fiber was assessed through an enzymatic-gravimetric method (AOAC 985.29). Available carbohydrates were estimated by weight difference.

Energy (kcal/100 g fw) was calculated using the Atwater general factors: 4 kcal/g for protein and (available) carbohydrates, 9 kcal/g for fat, and 2 kcal/g for dietary fiber [14].

#### 2.3.2. Soluble Sugars and Organic Acids

Soluble (free) sugars were extracted using a hydroethanolic solution, with melezitose added as internal standard, following established protocols [15]. The separation was achieved via high-performance liquid chromatography (HPLC) on a multimode column. Detection was carried out using a refractive index detector. Analytes were identified by matching retention times with reference standards, and their concentrations (g/100 g fw) were determined using the internal standard method.

Organic acids were extracted using a meta-phosphoric acid solution. Separation and quantification were conducted using ultra-fast liquid chromatography (UFLC) coupled with a photodiode array (PDA) detector, as outlined in a prior work [15]. A reverse-phase column was employed for chromatographic separation, with detection wavelengths set at 245 nm (ascorbic acid) and 215 nm (remaining organic acids). Quantification (mg/100 g fw) was achieved by comparing peak areas against the calibration curves in Table S3.

The sweetness index was calculated as the ratio of total soluble sugars to total organic acids.

#### 2.3.3. Fatty Acids and Tocopherols

The crude fat was subjected to transesterification to produce fatty acid methyl esters (FAMEs), which were subsequently analyzed by gas chromatography with flame ionization detection [15]. Identification was achieved by matching chromatographic retention times with a 37-component FAME reference standard. Results were expressed as relative percentages of individual fatty acids, with additional classification into saturated (SFA), monounsaturated (MUFA), and polyunsaturated (PUFA) categories.

Tocopherol analysis employed normal-phase HPLC with fluorescence detection (excitation 290 nm, emission 330 nm), using tocol as internal standard [15]. Compound identification was performed by retention time alignment with authentic standards, and quantification (mg/100 g fw) was conducted using the internal standard approach.

#### 2.3.4. Mineral Composition

Macrominerals (Na, K, Ca, Mg) and trace elements (Mn, Zn, Fe, Cu) were analyzed by atomic absorption spectroscopy (AAS). Approximately 0.25 g of each sample was submitted to microwave-assisted digestion (1600 W) with 10 mL of nitric acid, following a temperature ramp: heating to 200 °C for 20 min, increasing to 220 °C for 15 min, and holding at 220 °C for 20 min. After cooling, the digested solutions were diluted to 50 mL with deionized water. Specific pretreatments were applied before analysis: solutions were diluted in cesium chloride (1 g/L) for K and Na, and in lanthanum chloride (1 g/L) for Ca and Mg, while Mn, Cu, Fe, and Zn were analyzed directly without further dilution. Results were expressed in mg/100 g fw.

### 2.4. Preparation of Hydroethanolic Extracts

Approximately 1 g of powdered freeze-dried peel, pulp, and seed samples were subjected to a 1-h extraction, performed twice using 30 mL of an ethanol/water mixture (80:20, *v*/*v*) at room temperature. This solvent system was selected for its safety, widespread use in food-related applications, and proven effectiveness in extracting a broad range of phenolic compounds from plant materials. The extracts were filtered through Whatman No. 4 filter paper, then ethanol was removed using a rotary evaporator (150 mbar, 40 °C, 100 rpm), and the remaining aqueous phase was lyophilized to obtain dry extracts [15].

### 2.5. HPLC-DAD-ESI/MS Analysis of Phenolic Compounds

The dried extracts were reconstituted in an ethanol/water solution (20:80, *v*/*v*) at a concentration of 5 mg/mL, filtered through 0.22-μm membrane filters, and analyzed as described by Barros et al. [15]. Phenolic compounds were identified by matching their retention times and UV-Vis and mass spectral data to authenticated reference standards. When reference standards were unavailable, tentative identification was made by cross-referencing the obtained data with the literature reports. Quantification (mg/g extract) was performed by interpolating peak areas using calibration curves constructed from standards of chlorogenic acid, ferulic acid, sinapic acid, epicatechin, naringerin, and quercetin-3-*O*-glucoside (Table S3). For compounds lacking available standards, the calibration curve of the most structurally similar compound was used. Accordingly, results were expressed as mg equivalents of the closest standard per gram of extract.

### 2.6. Evaluation of Bioactive Properties

#### 2.6.1. In Vitro Cell-Based Antioxidant Activity

Working solutions were prepared by reconstituting the extracts in PBS (pH 7.4) or Tris–HCl buffer (20 mM, pH 7.4) for in vitro oxidative hemolysis inhibition (OxHLIA) and thiobarbituric acid reactive substances (TBARS) formation inhibition assays, respectively, and then serially diluted to different concentrations [16]. In OxHLIA, erythrocytes (2.8%, *v*/*v*) were incubated with the extract solutions (62.5–2000 µL/mL) and 2,2′-azobis(2-amidinopropane) dihydrochloride (160 mM) as a free radical generator, with hemolysis kinetics monitored at 690 nm. The TBARS assay involved incubating brain homogenate with the extract solutions (9.8–5000 μg/mL), FeSO_4,_ and ascorbic acid, followed by the addition of trichloroacetic and thiobarbituric acid (TBA) solutions, and the quantification of malondialdehyde (MDA)-TBA adducts at 532 nm. Trolox served as a positive control. Antioxidant activity was expressed as IC_50_ values (µg/mL) for OxHLIA (Δ*ts* of 60 and 120 min) and EC_50_ values (µg/mL) for TBARS inhibition.

#### 2.6.2. Antimicrobial Activity Against Foodborne Pathogens

The extracts reconstituted in 30% ethanol were used to evaluate minimum inhibitory (MIC) and fungicidal/bactericidal (MFC/MBC) concentrations (mg/mL) against foodborne pathogens (Table S1) using serial microdilution methods [17,18]. Sodium benzoate (E211) and potassium metabisulfite (E224) served as positive controls, with 30% ethanol as the negative control. After determining the MIC values (the lowest concentrations inhibiting visible microbial growth in 96-well plates), aliquots from negative-growth wells were subcultured in fresh medium to determine MBC/MFC values (the lowest concentrations achieving ≥99.5% microbial reduction).

### 2.7. Statistical Analysis

Results were presented as mean ± standard deviation (SD) from triplicate experimental measurements, except for antibacterial activity, which was reported as MIC, MFC, and MBC values. Means were rounded to match the precision of their SDs (one significant figure). Data normality (Shapiro–Wilk test) and variance homogeneity (Levene’s test) were verified before ANOVA. Based on homogeneity results, post hoc comparisons used either Tukey’s HSD (homoscedastic data) or Tamhane’s T2 test (heteroscedastic data). All analyses were performed at α = 0.05 using SPSS Statistics 22.0 (IBM Corp., Armonk, NY, USA).

Principal component analysis (PCA) was performed using OriginPro 2019b 9.6.5.169 (OriginLab Corp., Northampton, MA, USA). Prior to PCA, variables were auto-scaled (mean-centered and divided by their standard deviation) to ensure equal weighting. The PCA biplot was constructed using correlation matrix-based extraction, with loadings and scores projected on the first two principal components (PC1 and PC2). Variables were displayed as vectors to illustrate their contribution to sample discrimination, while different fruit parts (pulp, peel, seeds) were plotted as scores. Interpretation was based on the spatial distribution and alignment of variables and sample points within the biplot.

## 3. Results and Discussion

### 3.1. Nutritional Composition

Lulo, the fruit of *S. quitoense*, is primarily consumed as juices and smoothies due to its tangy and refreshing flavor. It is also used in the production of nectars, jams, ice creams, concentrates, and even fermented beverages and artisanal liqueurs. Therefore, it is essential to know the nutritional and chemical composition of its different parts, including the edible pulp and seeds, as well as the inedible but potentially valuable peel. This knowledge could contribute to more complete food composition tables and support the development of novel applications for this fruit.

#### 3.1.1. Centesimal Composition and Energy

Table 1 presents the nutritional composition of the different parts of *S. quitoense* fruit, including the overall composition of the edible portion estimated based on the specific composition and relative proportion of the pulp and seeds. The moisture content varied significantly between the fruit parts, ranging from 51 g/100 g in the seed to 87 g/100 g in the pulp. Gancel et al. reported a higher moisture content of 91.5 g/100 g for the edible portion of fruits cultivated in Ecuador [5], while the USDA FoodData Central reports 93 g/100 g [19]. Though slightly lower, the estimated moisture content of 82 g/100 g for the edible portion in this study remains within a comparable range.

Among the analyzed fruit parts, the seeds exhibited the highest contents of protein (4.37 g/100 g fw), crude fat (2.90 g/100 g fw), dietary fiber (19.4 g/100 g fw), and available carbohydrates (20.9 g/100 g fw). Conversely, the pulp had the lowest concentrations of these macronutrients. As a result, the seeds were also the most energy-dense part of the fruit, with 167 kcal/100 g fw, while the pulp contributed the least. For the edible portion, the calculated energy content was approximately 56 kcal/100 g fw. To the best of the authors’ knowledge, this is the first study reporting the nutritional composition of *S. quitoense* seeds.

Regarding the pulp, discrepancies were noted between the results in Table 1 and the USDA data. For instance, the protein content observed in this study (1.22 g/100 g fw) is notably higher than the 0.44 g/100 g fw reported by the USDA FoodData Central [19]. In terms of crude fat, the value obtained in this study (0.098 g/100 g fw) is lower than the 0.22 g/100 g fw reported by the USDA FoodData Central [18]. As for ash content, an indicator of total mineral content, this study reports 1.22 g/100 g fw, while 0.39 g/100 g fw is presented by the USDA FoodData Central [19]. Such differences might stem from edaphoclimatic conditions of the growing sites, agricultural practices, and post-harvest handling steps.

Carbohydrate content varied markedly among the different parts of the fruit, ranging from 5.9 to 20.9 g/100 g fw (Table 1), resulting in energy values ranging from 39 to 167 kcal/100 g fw. The edible portion contained 7.9 g/100 g of available carbohydrates and provided 56 kcal/100 g. In comparison, Obregón La Rosa and Lozano Zanelly reported 10.28 g/100 g fw of total carbohydrates and 45.35 kcal/100 g fw for whole fruits grown in Peru [4], while Acosta et al. found lower values (3.80 g/100 g fw of available carbohydrates and 18 kcal/100 g fw) in whole fruits from Costa Rica [20]. The USDA FoodData Central value for the carbohydrate content (5.9 g/100 g fw) in the pulp aligns closely with our results [19].

Dietary fiber emerged as a significant macronutrient in all parts of the fruit, particularly in the peel and seed, which contained 16.5 and 19.4 g/100 g fw, respectively. Notably, 100 g of dried peel provided approximately 57 g of dietary fiber, nearly ten times the minimum required for a product to be labeled “high in fiber” under European regulations [21]. According to Regulation (EC) No 1924/2006 of the European Parliament and the Council [21], the edible portion of *S. quitoense* can be classified as fat-free (<0.5 g/100 g) and high in dietary fiber (>6 g/100 g). When compared to the USDA FoodData Central value (1.1 g/100 g fw) [19], the dietary fiber content obtained in this study (4.5 g/100 g) appears significantly higher. In addition to natural variations in composition, this discrepancy may be due to differences in fiber quantification methodologies (e.g., enzymatic-gravimetric vs. AOAC variants), inclusion of mucilage or seed fragments in the pulp sample, or other differences in sample processing. Furthermore, fruit maturity stage at harvest or pre-treatment treatments like drying can concentrate fibrous material, influencing final results.

These findings highlight the potential of *S. quitoense* fruit peel as a valuable by-product for dietary fiber enrichment in food products. The incorporation of such fiber-rich by-products into novel formulations aligns with current trends in functional food development, contributing not only to improved consumer health but also to the reduction in food waste through the valorization and upcycling of agri-food by-products into value-added ingredients [22].

The relatively low energy value of the pulp (<100 kcal/100 g fw) reinforces its suitability as a component for low-calorie diets [19,23].

#### 3.1.2. Soluble Sugars and Organic Acids

The interplay between sugars and organic acids in fruits is fundamental to their overall appeal, influencing both taste and nutritional quality. While simple sugars like glucose, fructose, and sucrose provide natural sweetness and serve as an energy source, organic acids such as citric and ascorbic acids impact distinctive tartness, enhance stability, and offer health-promoting properties. Beyond their immediate impact on flavor, these compounds also influence shelf-life and dietary benefits, ultimately affecting consumer preferences and market demand [24].

As shown in Table 1, three soluble sugars were identified in the *S. quitoense* fruit samples. Fructose was most abundant in the peel and seed samples, while sucrose predominated in the pulp. The seeds exhibited the highest soluble sugar content (6.2 g/100 g fw), followed by the pulp (5.8 g/100 g fw) and the peel (4.5 g/100 g fw). In the edible portion, individual sugar concentrations were 2.64 g/100 g fw of sucrose, 2.23 g/100 g fw of fructose, and 0.97 g/100 g fw of glucose. Glucose was the least abundant and showed relatively consistent levels across all fruit parts. This sugar profile contrasts with that reported by Gancel et al., who found approximately 1.47 g/100 g fw of glucose, 1.31 g/100 g fw of sucrose, and 1.01 g/100 g fw of fructose for the edible part (values recalculated to fresh weight using moisture data provided by the author) [5]. Likewise, Acosta et al. reported sugar levels of 1.6 g/100 g fw for sucrose, 0.7 g/100 g fw for fructose, and 0.68 g/100 g fw for glucose in the edible portion of fruit from Costa Rica. Such variations are likely due to differences in genotype, cultivation practices, and edaphoclimatic conditions, all of which can influence fruit metabolism and composition.

Regarding organic acids, citric acid was the predominant compound in all fruit parts, particularly concentrated in the pulp (4.22 g/100 g fw), consistent with the fruit’s notably sour taste (Table 1). The seeds contained only citric and oxalic acids, while the peel had higher concentrations of quinic and oxalic acids and was the only sample where shikimic acid was detected and quantified. Total organic acids in the pulp reached 4.74 g/100 g fw, exceeding the 2.69 g/100 g reported by Gancel et al. for *S. quitoense* fruit juice analyzed using HPLC with electrochemical detection [5].

Ascorbic acid (vitamin C), which is essential for collagen synthesis, iron absorption, and antioxidant defense [12], was most concentrated in the peel (25.2 mg/100 g fw), followed by the pulp (9.7 mg/100 g fw). These values are markedly higher than those reported by Gancel et al. for samples from Pichincha, Ecuador, who found 6.5 mg/100 g fw in the peel, 7.5 mg/100 g fw in the flesh (mesocarp and endocarp), and 11.5 mg/100 g fw in the placental tissue (values recalculated from dry weight using moisture data provided by the author) [5]. Based on the weight percentage of placental tissue and flesh reported in that study, the ascorbic acid content in the edible portion was estimated at 10.1 mg/100 g fw. In the present study, the edible portion contained approximately 8.4 mg/100 g fw, surpassing the values reported by USDA Food Data Central (3.2 mg/100 g fw) [19] and Sánchez-Capa et al. (4.16 mg/100 g fw) [25]. This amount represents approximately 9–10% of the reference dietary intake (RDI) for adult males and females [26]. Another study highlighted a considerably higher concentration of 30.1 mg/100 g fw of vitamin C [4], though this was estimated using 2,6-dichloroindophenol titration, a method that is prone to overestimating ascorbic acid content due to its low specificity.

In addition to sugar and acid content, other related physicochemical attributes further define fruit quality. A 100 g portion of *S. quitoense* fruit has been reported to possess a titratable acidity of 2.63 g citric acid equivalents and a total soluble solids content of 9.1° Brix [20]. These parameters are essential not only for flavor perception but also for post-harvest stability [20]. Herein, the sweetness index of the edible portion was calculated as 1.5, which is considerably lower than values reported for other fruits in the Solanaceae family, such as *Physalis peruviana* L. (7.1) [27]. This index falls below the threshold of 5 commonly used to characterize fruits as predominantly sweet-sour [28], confirming the acidic flavor profile of *S. quitoense* fruit.

#### 3.1.3. Tocopherols and Fatty Acids

Tocopherols, a group of vitamin E compounds, play a crucial role in human health due to their potent antioxidant properties. These compounds help protect cellular components from oxidative damage and support immune function, contributing to the prevention of cardiovascular and neurological diseases [29]. In addition to their physiological benefits, tocopherols have important applications in food systems, where they help retard lipid oxidation, thereby extending shelf life and preserving nutritional quality. Their dual role as essential nutrients and natural preservatives underscores their value in both nutrition and food technology [29].

The tocopherol composition varied among the different parts of *S. quitoense* fruit (Table 1). In the peel, both α- and δ-tocopherols were detected, while α- and γ-tocopherols were found in the pulp and seeds. The peel exhibited the highest total tocopherol content (13.0 g/100 g fw), with 7.9 mg/100 g fw of α-tocopherol and 5.12 mg/100 g fw of δ-tocopherol. The seeds were particularly rich in γ-tocopherol (1.7 mg/100 g fw), the predominant isomer in this tissue. In the pulp, α-tocopherol and γ-tocopherol were present at 0.37 mg/100 g fw and 0.197 mg/100 g fw, respectively, values somewhat comparable to those reported by the USDA FoodData Central (0.75 mg and 0.2 mg per 100 g, respectively), which does not report the presence of δ- or γ-tocopherols [19].

The high tocopherol content in *S. quitoense* fruit, particularly in the peel, highlights its potential as a valuable source of vitamin E. The distribution of tocopherol isomers across the different fruit parts contributes to antioxidant intake and enhances oxidative stability, which can benefit both nutritional quality and storage potential.

Table 2 presents the fatty acid composition of *S. quitoense* fruit parts. SFA were predominant in the peel and seeds, PUFA were most abundant in the pulp, and MUFA prevailed in the seeds. Palmitic acid (C16:0) was the main SFA across all parts, ranging from 24.0% in the pulp to 28.0% in the seeds. Notably, the pulp stood out for its high PUFA percentage (52.2%), mainly due to significant levels of α-linolenic acid (C18:3*n*-3, 28.1%) and linoleic acid (C18:2*n*-6, 23.9%), which are essential fatty acids known for their cardioprotective and anti-inflammatory effects. The high PUFA/SFA ratio (1.27) further underscores the pulp’s nutritional relevance.

In contrast, MUFA levels were highest in the seeds (60.7%), largely due to oleic acid (C18:1*n*-9c, 44.9%), a fatty acid associated with reduced inflammation. This profile is comparable to that of other edible oil-rich seeds, suggesting that *S. quitoense* seeds could serve as a valuable source of functional lipids. The peel also exhibited a notable MUFA proportion (26.1%), mainly due to oleic acid (19.9%) and nervonic acid (C24:1, 3.5%), the latter known for its relevance to neural development and cognitive function [30,31].

These findings are in line with those of Loizzo et al., who identified palmitic, oleic, and α-linolenic acids as major fatty acids in the peel and pulp with seeds of *S. quitoense* [32]. However, in the present study, the seeds showed a higher oleic acid content than previously reported, along with the presence of elaidic acid (C18:1*n*-9t), a long-chain fatty acid not reported in earlier studies. These differences may reflect genetic variations and environmental influences or be related to the extraction methods used [33].

Overall, the fatty acid and tocopherol profiles of *S. quitoense* fruit samples reveal its potential as a source of bioactive lipids and antioxidant vitamins, with the PUFA-rich pulp and MUFA-rich seeds offering both nutritional and functional benefits. Future research may focus on the extraction efficiency, oxidative stability, and functional applications of these compounds in food and nutraceutical products.

#### 3.1.4. Mineral Composition

Minerals are essential micronutrients involved in a wide array of physiological functions that support human health and well-being. They contribute to bone formation (e.g., Ca), oxygen transport (Fe), nerve function (Na, K, Mg), and serve as cofactors in numerous enzymatic reactions (Zn, Cu, Mn) [34]. Adequate and balanced intake of these elements is therefore crucial for maintaining metabolic homeostasis, preventing nutritional deficiencies, and reducing the risk of chronic diseases [34].

Among the studied fruit parts of *S. quitoense*, the peel exhibited the highest mineral content, a finding consistent with its elevated ash concentration (Table 3). K was the most abundant mineral in all samples, with concentrations of 899 mg/100 g in the peel, 459 mg/100 g in the pulp, and 403 mg/100 g in the seeds. These values significantly exceed the concentration reported by the USDA FoodData Central (200 mg/100 g fw) for the pulp [19] or by Obregón La Rosa and Lozano Zanelly (354.9 mg/100 g fw) for the whole fruit [4]. A 100 g serving of the edible fruit portion provides approximately 13% of the RDI for K for both adults [35], underscoring its value as a daily source of this essential electrolyte.

As shown in Table 3, Mg and Ca followed in abundance. The seeds had the highest Mg content (96 mg/100 g fw), while the peel had the highest Ca concentration (35 mg/100 g fw). The Mg levels found for the edible portion (29 mg/100 g fw) are consistent with the 25.1 mg/100 g fw previously reported for whole fruits from Peru, and higher than the 11 mg/100 g fw reported for the pulp [19]. In turn, the Ca content (5.4 mg/100 g fw) was lower than the previously reported 8 mg/100 g fw [19] and 15.7 mg/100 g fw [4]. These discrepancies highlight the influence of environmental and agronomic factors on mineral accumulation in plant tissues. Both elements are essential for muscle contraction, nerve signaling, and/or bone health [34]. Moreover, the presence of these minerals underscores the functional potential of the fruit’s inedible part.

The peel and especially the seeds also contained appreciable levels of Fe (1.30 and 2.08 mg/100 g fw, respectively), Mn (0.38 and 1.25 mg/100 g fw, respectively), and Zn (0.68 and 3.5 mg/100 g fw, respectively). Although a previous study reported a higher Fe content (3.46 mg/100 g fw) for whole fruits [4], the USDA FoodData Central reports a value (0.35 mg/100 g fw) comparable to that found in the edible pulp in the present study [18]. Moreover, the same previous study reported Mn (0.11 mg/100 g fw), Cu (0.12 mg/100 g fw), and zinc (0.17 mg/100 g fw) levels similar to those observed in the pulp [4]. The relatively high Fe content, particularly in the seeds, suggests that this fruit could help mitigate Fe deficiency anemia, especially among populations with limited access to animal-based sources of bioavailable Fe.

The mineral-rich composition of *S. quitoense*, particularly the peel and seeds, positions these fruit parts as promising candidates for valorization in the development of functional food and nutraceutical ingredients, among other valuable products. Such utilization would enhance the micronutrient profile of food products and promote sustainability by reducing agri-food waste and encouraging the circular use of plant-based resources, thus promoting more circular and smart food systems [4].

### 3.2. Phenolic Composition

The analysis of phenolic compounds in *S. quitoense* fruit extracts was conducted using HPLC-DAD-ESI/MS*^n^*, based on retention times (min), maximum UV-Vis absorption wavelengths (nm), deprotonated molecule ([M-H]^−^/[M]^+^), and main fragment ions in tandem MS^2^ for compound tentative identification, and calibration curves constructed with the most similar standards for quantification. These results are presented in Table 4, while representative chromatograms are shown in Figure S1 in the Supplementary Materials. The secondary metabolites identified in this work have been previously described in the biosynthetic pathways of the order Solanales [36].

As shown in Table 4, a total of 25 phenolic compounds were detected in the *S. quitoense* samples, comprising 7 phenolic acids, 13 flavonoids, and 5 HCAA (spermidine derivatives). Compound **1** ([M-H]^−^ at *m*/*z* 341 and main MS^2^ fragment ion at *m*/*z* 179 (due to the loss of a sugar moiety) was tentatively identified as caffeic acid hexoside [37,38]. Compound **2** showed the molecular ion [M-H]^−^ at 353 *m*/*z*, and the main fragment at *m*/*z* 191 (quinic acid) from the loss of a sugar moiety, followed by a relatively abundant fragment ion at *m*/*z* 179. This compound was tentatively identified as 3-*O*-caffeoylquinic acid [5,37,38]. Compounds **3** and **4** also showed their [M-H]^−^ at 353 *m*/*z*, with main fragments at *m*/*z* 191. However, the fragments at *m*/*z* 179 showed lower abundance, leading to the identification as *cis*- and *trans*-5-*O*-caffeoylquinic acids, respectively [5,37,38]. Compound **5** presented a deprotonated ion [M-H]^−^ at *m*/*z* 289, with ion fragments at *m*/*z* 159, 175, and 131, and maximum absorbance at 276 nm. It was tentatively identified as epicatechin [39]. Compound **6** showed a deprotonated molecule [M-H]^−^ at *m*/*z* 355, with the main fragment at *m*/*z* 193 from the loss of a sugar moiety, and maximum absorbance at 328 nm, being tentatively identified as ferulic acid hexoside. Compound **7** ([M-H]^−^ at *m*/*z* 385) exhibited its fragment ions at *m*/*z* 223 (due to sugar moiety loss), 207, and 161, with maximum absorbance at 331 nm, being tentatively identified as sinapic acid hexoside. Compound **8** was tentatively identified as ferulic acid dihexoside ([M-H]^−^ at *m*/*z* 517, with the main fragment at *m*/*z* 193, from the loss of two sugar moieties, and maximum absorbance at 325 nm). To the best of the authors’ knowledge, this is the first time that sinapic acid, ferulic acid, or their derivatives have been identified in the fruit tissues of *S. quitoense*.

Compound **9** was tentatively identified as isorhamnetin-*O*-dihexoside due to its deprotonated molecule [M-H]^−^ at *m*/*z* 639, fragment ions at *m*/*z* 477 and 315 (isorhamnetin aglycone), each from the loss of one sugar moiety [5]. Compound **10** was tentatively identified as quercetin-*O*-hexosyl-deoxyhexosyl-hexoside, due to its deprotonated molecule [M-H]^−^ at *m*/*z* 771, fragments at *m*/*z* 609 (loss of a sugar moiety), and 301 (quercetin aglycone) [5]. Compound **11** showed its deprotonated molecule [M-H]^−^ at *m*/*z* 625, with fragment ions at *m*/*z* 463 (from the loss of a sugar moiety) and 301 (quercetin aglycone), leading to its identification as quercetin-*O*-dihexoside (MS-DAD). Compound **12** showed its deprotonated molecule [M-H]^−^ at *m*/*z* 755, with fragment ions at *m*/*z* 591 (from the loss of a sugar moiety) and 301 (quercetin aglycone), thus tentatively identified as quercetin-dideoxyhexosyl-hexoside [40]. Compound **13** was tentatively identified as isorhamnetin-*O*-dihexoside, due to its deprotonated molecule [M-H]^−^ at *m*/*z* 639 and fragment ions at *m*/*z* 477 (from the loss of sugar moiety) and 315 (isorhamnetin aglycone) [5]. Compound **14** showed a deprotonated ion [M-H]^−^ at *m*/*z* 609, with the main fragment ion at *m*/*z* 301 (quercetin aglycone) from the loss of a sugar moiety, leading to the tentative identification as quercetin-3-*O*-deoxyhexosyl-hexoside [5]. Compound **15** was tentatively identified as kaempferol-3-*O*-dideoxyhexosyl-hexoside [40] ([M-H]^−^ at *m*/*z* 739, fragment ions at *m*/*z* 575 from the loss of a sugar moiety, and *m*/*z* 285 from kaempferol aglycone, with maximum absorbance at 341 nm). Compound **16** was tentatively identified as isorhamnetin-*O*-deoxyhexosyl-deoxyhexosyl-hexoside, due to its deprotonated molecule [M-H]^−^ at *m*/*z* 769 and main fragment ion at *m*/*z* 315 (isorhamnetin aglycone) [41]. Compound **17** was tentatively identified as naringerin dihexoside due to its deprotonated ion [M-H]^−^ at *m*/*z* 595 and fragment ions at *m*/*z* 433 (loss of a sugar moiety) and 271 (naringerin aglycone). To the best of the authors’ knowledge, this is the first report identifying naringenin in the fruit tissues of *S. quitoense*.

For compounds **18** and **21**, the deprotonated molecule [M-H]^−^ appeared at *m*/*z* 799, with main fragment ions at *m*/*z* 637 (loss of a sugar moiety), and maximum absorbances at 326 nm. These were tentatively identified as *N1*,*N4*,*N8*-tris(dihydrocaffeoyl) spermidine hexosides [5] Compounds **19** and **23** were tentatively identified as *N1*,*N4*,*N8*-tris(dihydrocaffeoyl) spermidines because of their deprotonated molecule [M-H]^−^ at *m*/*z* 637, main fragment at *m*/*z* 473, and maximum absorbances at 327 nm [5].

Compound **20** was identified as quercetin-3-*O*-rutinoside due to its molecular ion [M-H]^−^ at *m*/*z* 609 and main ion fragment at *m*/*z* 301 (quercetin aglycone) [5]. Compound **22** was tentatively identified as kaempferol-3-*O*-deoxyhexosyl-hexoside due to its deprotonated molecule [M-H]^−^ at *m*/*z* 593, fragment ions at *m*/*z* 285 (kaempferol aglycone) and 429, and maximum absorbance at 321 nm [5]. Compound **24** showed its deprotonated ion [M-H]^−^ at *m*/*z* 623, with the main ion fragment at *m*/*z* 315 (isorhamnetin aglycone); it was tentatively identified as isorhamnetin-*O*-deoxyhexosyl-hexoside [5].

Compound **25** was tentatively identified as *N1*,*N4* or *N4*,*N8*-bis(dihydrocaffeoyl) spermidine due to its deprotonated molecule [M-H]^−^ at *m*/*z* 473, fragments at *m*/*z* 351 and 308, and maximum absorbance at 328 nm [5]. All these spermidine derivatives have been previously described in *S. quitoense* tissues [11].

The phenolic composition of the studied *S. quitoense* fruit parts varied significantly, with the seeds exhibiting the highest content of phenolic compounds (62.3 mg/g extract), followed by the peel (28.1 mg/g) and the pulp (20.9 mg/g) extracts. The seeds were particularly rich in HCAA, specifically spermidine derivatives, which accounted for 37.8 mg/g extract. Among these, *N1*,*N4* or *N4*,*N8*-bis(dihydrocaffeoyl) spermidine was the predominant compound, with a concentration of 10.7 mg/g extract. These findings are consistent with previous reports that identified spermidine derivatives in *S. quitoense* fruit, though they did not evaluate their distribution across fruit tissues [23].

Phenolic acids were also present in considerable amounts, with *trans*-5-*O*-caffeoylquinic acid emerging as the most abundant in both the pulp (12.1 mg/g extract) and the peel (8.6 mg/g extract), while its concentration in the seeds was markedly lower (1.15 mg/g extract). This distribution pattern is consistent with findings by Gancel et al., who reported chlorogenic acids primarily in the flesh and placental tissues of *S. quitoense* fruit [5].

Flavonoids were predominantly found in the peel (14.2 mg/g extract), with significantly lower concentrations in the pulp (1.88 mg/g) and seeds (0.20 mg/g). The major flavonoids identified in the peel were quercetin, isorhamnetin, and kaempferol derivatives, which are widely recognized for their antioxidant and antimicrobial activities [42,43]. Previous studies have also documented these compounds in *S. quitoense* peel extracts, particularly those obtained through acetone/water extractions [5]. The presence of flavonoids in the peel suggests that this often-discarded fruit fraction could be valorized for functional food applications.

The pulp was notably rich in ferulic acid hexoside (1.69 mg/g extract) and sinapic acid hexoside (1.70 mg/g extract), which were found in even higher concentrations in the seeds (7.1 mg/g and 7.0 mg/g extract, respectively). To the best of the authors’ knowledge, this is the first time that sinapic acid, ferulic acid, or their derivatives have been identified in *S. quitoense* fruit tissues. These compounds have been extensively reported as potent antioxidants with anti-inflammatory and antimicrobial properties [44,45], further highlighting the potential of *S. quitoense* seeds for developing bioactive food ingredients.

Additionally, naringenin dihexoside was detected in the peel (1.20 mg/g extract), marking the first report of this flavanone in *S. quitoense* fruit. This compound is of particular interest due to its potential anti-inflammatory and cardioprotective effects [46], which have been reported in other Solanaceae fruits, such as tomato and pepper [47,48]. The presence of naringenin in this species further expands the knowledge of its phytochemical profile and potential functional properties.

When comparing these results with previous studies, this study provides a more detailed characterization of the compounds’ localization in the fruit, confirming that spermidine derivatives are predominantly found in the seeds. Overall, these findings underscore the distinctive phytochemical composition of each *S. quitoense* fruit part, demonstrating that the seeds are an exceptionally rich source of bioactive phenolamides, while the peel and pulp are primarily composed of flavonoids and phenolic acids, respectively. This differentiation is important for guiding the industrial application of *S. quitoense* fruit by-products, particularly in the development of functional food ingredients, nutraceuticals, and antioxidant-rich beverages. Future research should focus on evaluating the bioavailability of these compounds and exploring their stability and synergistic effects in food formulations to maximize their health benefits.

### 3.3. Bioactive Properties

#### 3.3.1. In Vitro Antioxidant Activity

A diet rich in antioxidant-rich foods such as fruits and vegetables plays a vital role in cellular protection and the prevention of chronic diseases. Antioxidants such as polyphenols, vitamins C and E, and other bioactive compounds can neutralize free radicals and reactive species, thereby reducing oxidative stress [12,49,50]. This protective effect has been associated with a lower risk of cardiovascular diseases, neurodegenerative disorders, and metabolic imbalances, while also supporting immune function and maintaining cellular integrity [51].

The results regarding the antioxidant activity of *S. quitoense* fruit extracts are detailed in Table 5. In the OxHLIA assay, the results are expressed as IC_50_ values, representing the extract concentration required to protect 50% of erythrocytes from oxidative hemolysis for time intervals (Δ*t*) of 60 and 120 min. For the TBARS assay, EC_50_ values indicate the extract concentration needed to inhibit the formation of TBARS, such as MDA, by 50%. Thus, lower IC_50_ or EC_50_ values indicate higher antioxidant activity.

The OxHLIA results demonstrated that hydroethanolic extracts from all fruit parts exhibited antioxidant activity by effectively protecting erythrocytes from oxidative hemolysis (Table 5). At a 60 min Δ*t*, extract concentrations required to achieve 50% protection ranged from 202 µg/mL (for the peel) to 229 µg/mL (for the pulp). As expected, higher concentrations were necessary to maintain protection over a more extended period (120 min Δ*t*), with IC_50_ values of 325 µg/mL for the peel, 381 µg/mL for the pulp, and 360 µg/mL for the seeds. These findings suggest that the peel extract was the most effective in sustaining erythrocyte protection over time, likely due to its elevated content of bioactive compounds.

Similarly, the TBARS formation inhibition assay demonstrated that the peel and pulp extracts exhibited significantly higher antioxidant activity (IC_50_ ≈ 23.2 µg/mL) compared to the seed extract (IC_50_ = 107 µg/mL). This indicates that lipid peroxidation in porcine brain tissue was more effectively inhibited by the peel and pulp extracts, underscoring their potential to delay oxidative reactions in food systems.

A previous study has shown that incorporating *S. quitoense* whole fruit into bread enhances its antioxidant activity and nutritional value, as evidenced by increased levels of total phenolics, total carotenoids, and Trolox equivalent antioxidant capacity (TEAC) compared to control bread [2]. Additionally, dried *S. quitoense* fruit bagasse powder has demonstrated antioxidant activity, as evidenced by its effectiveness in DPPH and ABTS radical-scavenging activity. The drying and milling process stabilized the bagasse, preserving its bioactive compounds and supporting its use as a functional food ingredient with potential antioxidant benefits [52].

These findings confirm that *S. quitoense* fruit extracts possess notable antioxidant effects against oxidative hemolysis and especially against lipid peroxidation. This activity could be attributed to the presence of various bioactive compounds, including phenolic acids [53], phenolamides [54], and organic acids such as ascorbic acid [12]. The strong antioxidant activity of the peel extract supports its suitability for functional food and nutraceutical applications, reinforcing the potential of repurposing this valuable by-product.

#### 3.3.2. Antimicrobial Activity

Antimicrobial food ingredients are critical for ensuring food quality and safety, as well as protecting public health. Pathogens such as *Salmonella* spp., *Escherichia coli*, *Listeria monocytogenes*, and *Aspergillus* spp. are common food contaminants associated with foodborne illnesses and mycotoxin-related disorders. The use of antimicrobial agents helps inhibit microbial growth, reduces spoilage, and extends product shelf life. In this context, natural antimicrobials such as bioactive plant extracts have emerged as promising alternatives to some artificial additives, aligning with sustainable food production practices and growing consumer demand for clean-label products.

Table 5 presents the MIC and MBC/MFC values of *S. quitoense* fruit extracts against the selected foodborne pathogens. The extracts demonstrated selective and promising antimicrobial activity, with notable variations among fruit parts. Pulp and seed extracts displayed the strongest antibacterial effects, while the seed extract showed the most pronounced antifungal activity. The pulp extract presented the lowest MIC and MBC values, particularly against *L. monocytogenes* and *E. cloacae*, with results comparable to those obtained with standard food preservatives, E211 and E224 (Table 5). This suggests strong potential for its application as a natural antimicrobial agent. The seed extract also demonstrated considerable antibacterial activity, notably against *L. monocytogenes*, along with moderate inhibition of *S. aureus* and *E. coli*.

Regarding antifungal activity, the seed extract was the most effective, especially against *A. versicolor*, outperforming the reference preservative E211. It also showed strong inhibitory activity against *A. niger* and *T. viride*, whereas peel and pulp extracts were generally less effective. None of the extracts exhibited significant activity against *A. fumigatus* or *P. verrucosum* var. *cyclopium*, and only limited inhibition was observed against *P. funiculosum*, for which a MIC of 12 mg/mL was recorded for the peel extract, while pulp and seed extracts showed no activity at the tested concentrations. These results indicate a relatively narrow antifungal spectrum toward these mold species.

The antimicrobial potential of *S. quitoense* is supported by previous research. For instance, Tenea et al. isolated a *Weissella cibaria* strain from wild *S. quitoense* fruit capable of producing an antimicrobial peptide effective against *S. enterica* and *E. coli* [55], while Jain et al. reported notable antibacterial effects of methanolic fruit extracts against *P. aeruginosa* [56]. The seed extract’s efficacy may be partially attributed to its spermidine derivatives, which have been reported to disrupt fungal polyamine metabolism [8], explaining its pronounced effects against *L. monocytogenes*, *A. versicolor*, *A. niger*, and *T. viride*.

Overall, these findings highlight the pulp and seed fractions of *S. quitoense* as promising sources of natural antimicrobials. Their incorporation into functional food systems could contribute to reducing the reliance on artificial additives, supporting the development of clean-label and sustainable food preservation strategies.

### 3.4. Multivariate Analysis

**Figure 2:**
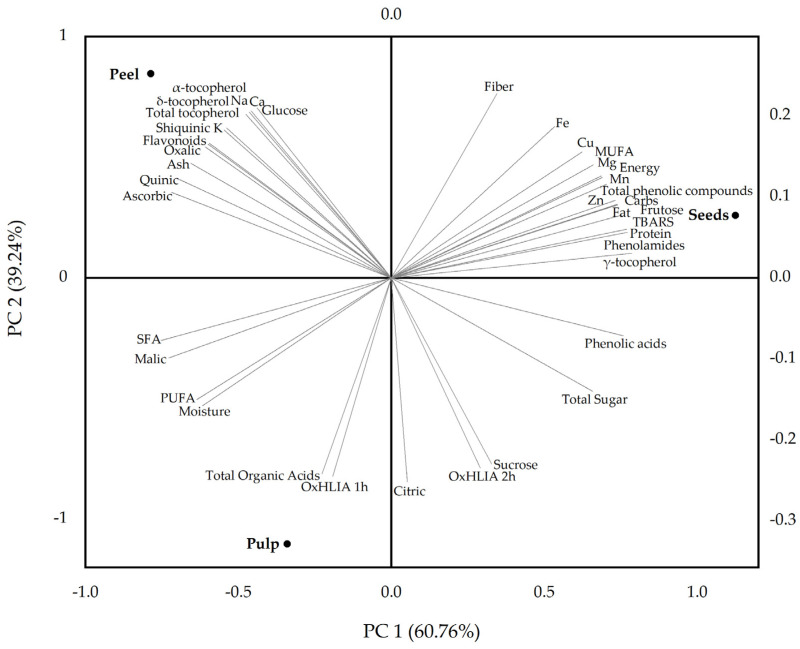
Principal component analysis (PCA) biplot showing the distribution of pulp, peel, and seed samples from *Solanum quitoense* fruit based on their nutritional and phytochemical composition and in vitro antioxidant activity.

The PCA biplot (Figure 2) provides a summarized visualization of the distribution patterns among the different fruit parts of *S. quitoense* based on their nutritional and phytochemical composition and in vitro antioxidant activity. The first two principal components (PC1 and PC2) cumulatively explain 100% of the total variance, with PC1 accounting for 60.76% and PC2 for 39.24%, ensuring a reliable and informative representation of the multivariate data.

The analysis showed that pulp and peel exhibit compositional similarity along PC1 but are separated along PC2. In contrast, the seeds were clearly distinct along PC1, demonstrating a significantly different composition compared to pulp and peel, with higher contents of proteins, lipids, and phenolic compounds. The pulp was strongly associated with higher levels of total organic acids, citric acid, sucrose, moisture, and PUFA, and exhibited a lower oxidative hemolysis inhibition capacity. The peel stood out for its elevated content of α- and δ-tocopherols, total tocopherols, quinic acid, ascorbic acid, glucose, macrominerals (Na, Ca, K), and flavonoids. The seeds contained the highest concentrations of proteins, crude fat, carbohydrates, fructose, γ-tocopherol, MUFA, trace elements (Zn, Cu, Mn, Fe), and phenolic compounds, particularly phenolamides. The TBARS formation inhibition capacity of the seed extract was lower than that of the other fruit parts.

Overall, these findings highlight the potential for targeted valorization of each fruit part, from antioxidant-rich peel extracts to protein- and polyphenol-enriched seed fractions. Such differentiation aligns with circular economy principles, promoting sustainable utilization of *S. quitoense* fruit in the development of functional foods and nutraceuticals, among other consumer goods.

## 4. Conclusions

This study revealed distinct nutritional and bioactive profiles in *S. quitoense* fruit parts, supporting their diverse applications. The seeds emerged as the most nutrient-dense, rich in protein, dietary fiber, γ-tocopherol, and spermidine derivatives, contributing to their antioxidant and antimicrobial potential. The pulp was characterized by its low energy value and high citric acid content and was well suited for use in functional food ingredients. In contrast, the peel, abundant in ascorbic acid and flavonoids, stood out as a natural source of antioxidants. The phenolic composition varied markedly among the fruit parts, with the seeds showing a high content of spermidine derivatives and the peel being rich in flavonoids. Antioxidant assays confirmed strong in vitro activity, particularly in the peel, while the seed extract showed notable antifungal effects.

These findings highlight the potential for valorizing *S. quitoense* edible pulp and by-products (peel and seeds) as functional ingredients for foods, nutraceuticals, and dietary supplements. Future research should focus on optimizing extraction techniques (including solvent selection and process intensification approaches), assessing the bioaccessibility and bioavailability of phenolic compounds, and exploring synergistic effects to fully harness their value in nutrition and health applications.

## Figures and Tables

**Table 1:** Centesimal composition, energy content, and composition of soluble sugars, organic acids, and tocopherols in *S. quitoense* fruit peel, pulp, seeds, and edible portion (pulp and seeds).

Constituent	Content (Per 100 g fw)			
	Peel	Pulp	Seeds	Edible Part *
Moisture (g)	71 ± 3 ^b^	87 ± 1 ^a^	51 ± 3 ^c^	82
Proteins (g)	2.06 ± 0.03 ^b^	1.22 ± 0.03 ^c^	4.37 ± 0.06 ^a^	1.64
Ash (g)	1.87 ± 0.03 ^a^	1.22 ± 0.06 ^b^	1.03 ± 0.06 ^c^	1.19
Crude fat (g)	0.49 ± 0.01 ^b^	0.098 ± 0.002 ^c^	2.90 ± 0.07 ^a^	0.48
Dietary fiber	16.5 ± 0.8 ^b^	4.5 ± 0.3 ^c^	19.4 ± 0.6 ^a^	6.5
Carbohydrates (g)	7.3 ± 0.1 ^b^	5.9 ± 0.4 ^c^	20.9 ± 0.4 ^a^	7.9
Energy (kcal)	77 ± 2 ^b^	39 ± 3 ^c^	167 ± 3 ^a^	56
Fructose (g)	2.2 ± 0.1 ^b^	2.11 ± 0.04 ^b^	3.0 ± 0.2 ^a^	2.23
Glucose (g)	1.0 ± 0.1 ^a^	0.97 ± 0.02 ^a^	0.98 ± 0.03 ^a^	0.97
Sucrose (g)	1.33 ± 0.04 ^c^	2.7 ± 0.1 ^a^	2.26 ± 0.04 ^b^	2.64
Total sugar (g)	4.5 ± 0.2 ^c^	5.8 ± 0.2 ^b^	6.2 ± 0.2 ^a^	5.9
Oxalic acid (mg)	134 ± 11 ^a^	60 ± 3 ^b^	49 ± 2 ^c^	68
Quinic acid (mg)	559 ± 43 ^a^	180 ± 4 ^b^	nd	156
Malic acid (mg)	220 ± 16 ^b^	272 ± 13 ^a^	nd	235
Shikimic acid (mg)	40 ± 2	nd	nd	-
Citric acid (g)	1.57 ± 0.02 ^c^	4.2 ± 0.3 ^a^	2.53 ± 0.06 ^b^	3.99
Ascorbic acid (mg)	25.2 ± 0.3 ^a^	9.7 ± 0.2 ^b^	nd	8.4
Total organic acids (g)	2.56 ± 0.09 ^b^	4.7 ± 0.3 ^a^	2.58 ± 0.06 ^b^	4.45
α-Tocopherol (mg)	7.9 ± 0.1 ^a^	0.37 ± 0.01 ^b^	0.25 ± 0.02 ^c^	0.35
β-Tocopherol (mg)	nd	nd	nd	nd
γ-Tocopherol (mg)	nd	0.197 ± 0.004 ^b^	1.7 ± 0.1 ^a^	0.4
δ-Tocopherol (mg)	5.12 ± 0.03	nd	nd	nd
Total tocopherols (mg)	13.0 ± 0.1 ^a^	0.57 ± 0.01 ^c^	2.0 ± 0.1 ^b^	0.76

Data are presented as mean ± SD (*n* = 3), and different superscript letters (a–c) within each row denote statistically significant differences between samples (*p* < 0.05), as determined by one-way ANOVA. * Average values calculated based on the weight percentage of pulp and seeds. nd—not detected.

**Table 2:** Fatty acid profiles of *S. quitoense* fruit peel, pulp, and seeds.

Fatty Acid	Peel (Relative %)	Pulp (Relative %)	Seeds (Relative %)
C13:0	0.51 ± 0.03	nd	nd
C14:0	1.07 ± 0.07 ^a^	0.45 ± 0.01 ^b^	0.30 ± 0.01 ^c^
C14:1	0.20 ± 0.01	nd	nd
C15:0	0.46 ± 0.04 ^a^	0.35 ± 0.01 ^b^	0.11 ± 0.01 ^c^
C16:0	25.7 ± 0.2 ^b^	24.0 ± 0.6 ^c^	28.0 ± 0.4 ^a^
C16:1	1.8 ± 0.1 ^b^	0.23 ± 0.01 ^c^	4.85 ± 0.05 ^a^
C17:0	0.52 ± 0.03 ^b^	0.70 ± 0.06 ^a^	0.55 ± 0.02 ^b^
C17:1	0.08 ± 0.01 ^b^	nd	0.25 ± 0.02 ^a^
C18:0	6.2 ± 0.2 ^b^	7.0 ± 0.4 ^a^	0.52 ± 0.03 ^c^
C18:1*n*-9c	19.9 ± 0.4 ^b^	6.6 ± 0.2 ^c^	44.9 ± 0.9 ^a^
C18:1*n*-9t	nd	nd	10.3 ± 0.5
C18:2*n*-6c	9.0 ± 0.1 ^b^	23.9 ± 0.6 ^a^	nd
C18:2*n*-6t	nd	0.16 ± 0.01	nd
C18:3*n*-6	nd	nd	0.48 ± 0.04
C18:3*n*-3	23.6 ± 0.3 ^b^	28.1 ± 0.4 ^a^	5.6 ± 0.1 ^c^
C20:0	2.08 ± 0.04 ^b^	2.49 ± 0.08 ^a^	1.08 ± 0.05 ^c^
C20:1	0.59 ± 0.02 ^a^	nd	0.37 ± 0.03 ^b^
C20:2	0.50 ± 0.02 ^b^	nd	0.30 ± 0.01 ^a^
C21:0	0.34 ± 0.01 ^b^	0.18 ± 0.01 ^a^	nd
C20:3*n*-3	0.42 ± 0.03	nd	nd
C22:0	1.46 ± 0.04 ^b^	2.20 ± 0.03 ^a^	0.95 ± 0.04 ^c^
C20:5*n*-3	0.18 ± 0.01	nd	nd
C23:0	0.90 ± 0.06 ^a^	0.52 ± 0.01 ^b^	0.34 ± 0.01 ^c^
C24:0	0.74 ± 0.01 ^c^	2.92 ± 0.06 ^a^	1.07 ± 0.08 ^b^
C24:1	3.5 ± 0.2	nd	nd
Fatty acid class			
SFA	40.2 ± 0.8 ^a^	40.98 ± 0.06 ^a^	32.9 ± 0.5 ^b^
MUFA	26.1 ± 0.4 ^b^	6.9 ± 0.2 ^c^	60.7 ± 0.4 ^a^
PUFA	33.7 ± 0.4 ^b^	52.2 ± 0.2 ^a^	6.42 ± 0.09 ^c^
PUFA/SFA	0.84 ± 0.02 ^b^	1.27 ± 0.01 ^a^	0.2 ± 0.01 ^c^

Data are presented as mean ± SD (*n* = 3), and different superscript letters (a–c) within each row denote statistically significant differences between samples (*p* < 0.05), as determined by one-way ANOVA. C13:0—Tridecanoic acid, C14:0—myristic acid, C14:1—myristoleic acid, C15:0—pentadecanoic acid, C16:0—palmitic acid, C16:1—palmitoleic acid, C17:0—heptadecanoic acid, C17:1—heptadecenoic acid, C18:0—stearic acid, C18:1n9c—oleic acid, C18:1*n*-9t—elaidic acid, C18:2*n*-6c—linoleic acid, C18:2*n*-6t—linolelaidic acid, C18:3*n*-6—γ-linolenic acid, C18:3*n*-3—α-linolenic acid, C20:0—arachidic acid, C20:1—eicosenoic acid, C20:2—eicosadienoic acid, C21:0—heneicosanoic acid, C20:3*n*-3—eicosatrienoic acid, C22:0—behenic acid, C20:5*n*-3—eicosapentaenoic acid, C23:0—tricosanoic acid, C24:0—lignoceric acid, C24:1—nervonic acid, SFS—saturated fatty acids, MUFA—monounsaturated fatty acids, PUFA—polyunsaturated fatty acids. nd—not detected.

**Table 3:** Mineral content in *S. quitoense* fruit peel, pulp, seeds, and edible portion (pulp and seeds).

Element	Content (Per 100 g fw)			
	Peel	Pulp	Seeds	Edible Part *
K (mg)	899 ± 30 ^a^	459 ± 11 ^b^	403 ± 20 ^c^	451
Na (mg)	10.8 ± 0.7 ^a^	4.0 ± 0.2 ^c^	4.9 ± 0.3 ^b^	4.1
Ca (mg)	35 ± 2 ^a^	4.8 ± 0.2 ^c^	9.1 ± 0.2 ^b^	5.4
Mg (mg)	42 ± 2 ^b^	18.8 ± 0.4 ^c^	96 ± 5 ^a^	29
Fe (mg)	1.30 ± 0.02 ^b^	0.26 ± 0.01 ^c^	2.08 ± 0.07 ^a^	0.51
Mn (mg)	0.38 ± 0.01 ^b^	0.079 ± 0.003 ^c^	1.25 ± 0.06 ^a^	0.24
Cu (mg)	0.25 ± 0.01 ^b^	0.124 ± 0.004 ^c^	0.42 ± 0.03 ^a^	0.16
Zn (mg)	0.68 ± 0.03 ^b^	0.18 ± 0.01 ^a^	3.5 ± 0.2 ^c^	0.63

Data are presented as mean ± SD (*n* = 3), and different superscript letters (a–c) within each row denote statistically significant differences between samples (*p* < 0.05), as determined by one-way ANOVA. * Average values calculated based on the weight percentage of pulp and seeds.

**Table 4:** Content of phenolic compounds tentatively identified in *S. quitoense* fruit peel, pulp, and seed extracts. The retention time (Rt), maximum absorption wavelengths in the UV-vis region (λ_max_), deprotonated molecule, and MS^2^ fragment ions (with relative abundance in brackets) are presented.

Peak	Rt (min)	λ_max_ (nm)	[M-H]^−^ (*m*/*z*)	MS^2^ (*m*/*z*)	Tentative Identification	Reference	Content (mg/g Extract)		
							Peel	Pulp	Seed
**1**	5.44	328	341	179(100), 135(12)	Caffeic acid hexoside	[37,38]	-	0.35 ± 0.01	-
**2**	6.76	326	353	191(100), 179(39)	3-*O*-Caffeoylquinic acid	[5,37,38]	1.57 ± 0.05 ^a^	0.67 ± 0.01 ^b^	0.53 ± 0.01 ^c^
**3**	7.03	326	353	191(100), 179(7)	*cis*-5-*O*-Caffeoylquinic acid	[5,37,38]	3.7 ± 0.2 ^a^	2.47 ± 0.04 ^b^	1.47 ± 0.02 ^c^
**4**	7.34	326	353	191(100), 179(12)	*trans*-5-*O*-Caffeoylquinic acid	[5,37,38]	8.60 ± 0.09 ^b^	12.1 ± 0.4 ^a^	1.15 ± 0.02 ^c^
**5**	9.02	276	289	159(100), 175(38), 131(20)	(-)-Epicatechin	[39]	-	1.00 ± 0.01 ^a^	0.202 ± 0.004 ^b^
**6**	9.20	328	355	193(100)	Ferulic acid hexoside	MS/DAD	-	1.69 ± 0.06 ^b^	7.1 ± 0.1 ^a^
**7**	9.73	331	385	223(100), 207(100), 161(23)	Sinapic acid hexoside	MS/DAD	-	1.70 ± 0.05 ^b^	7.0 ± 0.1 ^a^
**8**	10.38	325	517	193(100), 134(70), 178(40)	Ferulic acid dihexoside	MS/DAD	-	-	7.1 ± 0.1
**9**	11.19	349	639	477(100), 315(20)	Isorhamnetin-*O*-dihexoside	[5]	0.30 ± 0.01	-	-
**10**	13.68	343	771	609(100), 301(32)	Quercetin-*O*-hexosyl-deoxyhexosyl-hexoside	[5]	1.01 ± 0.01	-	-
**11**	14.23	342	625	463(100), 301(23)	Quercetin-*O*-dihexoside	MS/DAD	0.97 ± 0.01	-	-
**12**	14.56	354	755	591(100), 301(93)	Quercetin-*O*-dideoxyhexosyl-hexoside	[40]	2.19 ± 0.07 ^a^	0.88 ± 0.01 ^b^	-
**13**	15.43	356	639	477(56), 315(100)	Isorhamnetin-*O*-dihexoside	[5]	1.10 ± 0.01	-	-
**14**	16.18	353	609	301(100)	Quercetin-*O*-deoxyhexosyl-hexoside	[5]	1.45 ± 0.04	-	-
**15**	16.56	341	739	575(100), 285(40), 593(38)	Kaempferol-*O*-dideoxyhexosyl-hexoside	[40]	0.97 ± 0.01	-	-
**16**	16.97	351	769	315(100)	Isorhamnetin-*O*-deoxyhexosyl-deoxyhexosyl-hexoside	[41]	1.01 ± 0.02	-	-
**17**	17.17	284	595	433(100), 271(14)	Naringerin dihexoside	MS/DAD	1.20 ± 0.01	-	-
**18**	17.42	326	799	637(100)	*N1*,*N4*,*N8*-tris(dihydrocaffeoyl) spermidine hexoside	[5]	-	-	9.9 ± 0.2
**19**	17.66	327	637	473(100)	*N1*,*N4*,*N8*-tris(dihydrocaffeoyl) spermidine	[5]	-	-	6.66 ± 0.08
**20**	17.79	351	609	301(100)	Quercetin-3-*O*-rutinoside	[5]	1.28 ± 0.04	-	-
**21**	18.35	326	799	637(100)	*N1*,*N4*,*N8*-tris(dihydrocaffeoyl) spermidine hexoside	[5]	-	-	6.2 ± 0.1
**22**	18.57	321	593	285(100), 429(54)	Kaempferol-*O*-deoxyhexosyl-hexoside	[5]	1.33 ± 0.02	-	-
**23**	18.58	325	637	473(100)	*N1*,*N4*,*N8*-tris(dihydrocaffeoyl) spermidine	[5]	-	-	4.5 ± 0.1
**24**	19.03	351	623	315(100)	Isorhamnetin-*O*-deoxyhexosil-hexoside	[5]	1.41 ± 0.05	-	-
**25**	21.11	328	473	351(100), 308(88)	*N1*,*N4* or *N4*,*N8*-bis(dihydrocaffeoyl) spermidine	[4]	-	-	10.7 ± 0.3
					Σ Phenolic acids		13.9 ± 0.3 ^c^	19.0 ± 0.4 ^b^	24.3 ± 0.5 ^a^
					Σ Flavonoids		14.2 ± 0.4 ^a^	1.88 ± 0.01 ^b^	0.20 ± 0.01 ^c^
					Σ Phenolamides/HCAA/Spermidine derivatives		-	-	37.8 ± 0.1
					Σ Phenolic compounds		28.1 ± 0.7 ^b^	20.9 ± 0.4 ^c^	62.3 ± 0.4 ^a^

Data are presented as mean ± SD (*n* = 3), and different superscript letters (a–c) within each row denote statistically significant differences between samples (*p* < 0.05), as determined by one-way ANOVA. Compounds **1**–**4**, **18**, **19**, **21**, **23**, and **25** were quantified using chlorogenic acid standard. Compound **5** was quantified using epicatechin standard. Compounds **6** and **8** were quantified using ferulic acid standard. Compound **7** was quantified using sinapic acid standard. Compounds **9**–**16**, **20**, **22**, and **24** were quantified using quercetin-3-*O*-glucoside standard. Compound **17** was quantified using naringerin standard.

**Table 5:** Antioxidant, antibacterial, and antifungal activity of *S. quitoense* fruit peel, pulp, and seed extracts and positive controls.

Bioactivity		Peel Extract		Pulp Extract		Seed Extract		Positive Control			
Antioxidant activity								Trolox			
OxHLIA (IC_50_, μg/mL)	Δ*t* 60 min	202 ± 4 ^b^		229 ± 4 ^c^		204 ± 4 ^b^		19.6 ± 0.7 ^a^			
	Δ*t* 120 min	325 ± 4 ^b^		381 ± 8 ^c^		360 ± 12 ^c^		41 ± 1 ^a^			
TBARS formation inhibition (EC_50_, μg/mL)		23.8 ± 0.8 ^b^		22.4 ± 0.6 ^b^		107 ± 4 ^c^		5.4 ± 0.3 ^a^			
Antimicrobial activity								E211		E224	
Bacterial strains		MIC	MBC	MIC	MBC	MIC	MBC	MIC	MBC	MIC	MBC
*Bacillus cereus*		12.0	18.0	6.00	12.0	6.00	12.0	0.50	0.50	2.00	4.00
*Staphylococcus aureus*		12.0	24.0	6.00	12.0	12.0	18.0	4.00	4.00	1.00	1.00
*Listeria monocytogenes*		6.00	12.0	3.00	6.00	1.50	3.00	1.00	2.00	0.50	1.00
*Escherichia coli*		12.0	24.0	6.00	12.0	12.0	18.0	1.00	2.00	0.50	1.00
*Enterobacter cloacae*		12.0	18.0	3.00	6.00	6.00	12.0	2.00	4.00	0.50	0.50
*Salmonella enterica* subsp. *enterica* ser. Typhimurium		12.0	18.0	6.00	12.0	12.0	18.0	1.00	2.00	1.00	1.00
Fungal strains		MIC	MFC	MIC	MFC	MIC	MFC	MIC	MFC	MIC	MFC
*Aspergillus fumigatus*		≥24.0	≥24.0	≥24.0	≥24.0	≥24.0	≥24.0	1.00	2.00	1.00	1.00
*Aspergillus versicolor*		3.00	6.00	6.00	12.0	1.50	3.00	2.00	4.00	1.00	1.00
*Aspergillus niger*		12.0	18.0	12.0	18.0	3.00	6.00	1.00	2.00	1.00	1.00
*Trichoderma viride*		12.0	18.0	12.0	18.0	6.00	12.0	1.00	2.00	0.50	0.50
*Penicillium funiculosum*		12.0	24.0	≥24.0	≥24.0	≥24.0	≥24.0	1.00	2.00	0.50	0.50
*Penicillium verrucosum* var. *cyclopium*		≥24.0	≥24.0	≥24.0	≥24.0	≥24.0	≥24.0	2.00	4.00	1.00	1.00

For antioxidant activity, data are presented as mean ± SD (*n* = 3), and different superscript letters (a–c) within each row denote statistically significant differences between samples (*p* < 0.05), as determined by one-way ANOVA. MIC/MBC/MFC (mg/mL).

## Data Availability

The original contributions presented in this study are included in the article/Supplementary Materials, further inquiries can be directed to the corresponding authors.

## Supplementary Materials

The following supporting information can be downloaded at: https://www.mdpi.com/article/10.3390/foods14122083/s1, Table S1: chemicals, standards, and biological materials used in the analyses; Table S2: equipment used in the nutritional and chemical analysis; Table S3: calibration curves used in the quantification of organic acids and phenolic compounds; Figure S1: chromatograms of *Solanum quitoense* peel (a), pulp (b), and seeds (c) hydroethanolic extracts recorded at 280, 330, and 370 nm.
