# Supplementary material for: Nutrients, Phytochemicals, and In Vitro Antioxidant and Antimicrobial Activities of Lulo (Solanum quitoense Lam.) Fruit Pulp, Peel, and Seeds
Source: Foods. 2025 Jun 13;14(12):2083. doi: 10.3390/foods14122083 (PMC12191578; doi:10.3390/foods14122083)
Supplement: Supplementary file 1 [file foods-14-02083-s001.zip › foods-3672123-supplementary.pdf]

Supplementary Material

Nutrients, Phytochemicals, and In Vitro Antioxidant and Antimicrobial Activities of Lulo (*Solanum quitoense* Lam.) Fruit Pulp, Peel, and Seeds

Table S1. Chemicals, standards, and biological materials used in the analyses.

**Chemicals, standards, and biological material**	**Suppliers**
Solvents for chromatographic analysis	Merck, Darmstadt, Germany
Chlorogenic acid, ferulic acid, sinapic acid, epicatechin, naringering, and quercetin-3-*O*-glucoside (purity > 98%)	Extrasynthèse, Genay, France
Melezitose, α, β, γ, and δ tocopherols, oxalic, quinic, malic, ascorbic, citric, and fumaric acids, Supelco® 37-component FAME mix (47885-U), 6-Hydroxy-2,5,7,8-tetramethylchroman-2-carboxylic acid (Trolox), 2,2'-azobis(2-methylpropionamidine) dihydrochloride (AAPH), sodium benzoate (E211), and potassium metabisulfite (E224)	Sigma-Aldrich, St. Louis, MO, USA
Escherichia coli (ATCC 25922), *Enterobacter cloacae* (ATCC 35030), *Salmonella enterica* subsp. *enterica* serovar Typhimurium (ATCC 13311), *Staphylococcus aureus* (ATCC 11632), *Bacillus cereus* (food isolate), *Listeria monocytogenes* (NCTC 7973), *Aspergillus fumigatus* (ATCC 9197), *Aspergillus niger* (ATCC 6275), *Aspergillus versicolor* (ATCC 11730), *Penicillium funiculosum* (ATCC 36839), *Penicillium verrucosum* var. *cyclopium* (food isolate), and *Trichoderma viride* (IAM 5061)	Mycological Laboratory, Institute for Biological Research "Sinisa Stanković", University of Belgrade Serbia
K, Na, Ca, and Mg standard solutions	AnalytiChem, Zedelgem, Belgium
Mn, Zn, Fe, and Cu standard solutions	Panreac AppliChem, ITW reagents, Barcelona

Table S2. Equipment used in the nutritional and chemical analysis.

**Equipment**	**Suppliers**
PMB moisture analyzer	Adam Equipment, Kingston, Milton Keynes, UK
FreeZone 4.5 freeze-dryer	Labconco, Kansas City, MO, USA
Pro-Nitro-A Macro-Kjeldahl automatic distillation and titration unit	JP Selecta, Barcelona, Spain
PinAAcle 900T Spectrometer + furnace autosampler AS900	Perkin Elmer, Waltham, MA, USA
Knauer Smartline system 1000	Knauer, Berlin, Germany
Eurospher 100-5 NH2 column (250 mm × 4.6 mm, 5 μm)	Knauer, Berlin, Germany
Knauer Smartline 2300 RI detector	Knauer, Berlin, Germany
Shimadzu 20 A series UFLC system	Shimadzu Corporation, Tokyo, Japan
SphereClone RP C18 column (250 mm × 4.6 mm, 5 μm)	Phenomenex, CA, USA
YL 6500 GC-FID	Young In Chromass, Anyang, Korea
Zebron™ ZB-FAME capillary column (30 m × 0.25 mm, 0.20 μm)	Phenomenex, CA, USA
YMC-Pack Polyamine II column (250 mm × 4.6 mm, 5 μm)	YMC America, MA, USA
Jasco FP-2020 FL detector	Jasco, Tokyo, Japan
Dionex Ultimate 3000 HPLC	Thermo Scientific, San Jose, CA, USA
Waters Spherisorb® S3 ODS-2 C18 column (4.6 mm × 150 mm, 3 μm)	Waters, Milford, MA, USA
LTQ XL linear ion trap mass spectrometer	Thermo Finnigan, San Jose, CA, USA

Table S3. Calibration curves used in the quantification of organic acids and phenolic compounds.

Standard	Concentration range	Equation	r^2	Limit of detection (μg/mL)	Limit of quantification (μg/mL)
Oxalic acid	0.01-1.25 (mg/mL)	$y = 1E{+}07x + 231891$	0.9999	6.3	20.8
Quinic acid	0.08-10 (mg/mL)	$y = 671557x + 14583$	0.9998	11.3	37.6
Malic acid	0.08-10 (mg/mL)	$y = 950041x + 6255{,}6$	0.9999	15.9	52.9
Ascorbic acid	0.008-1 (mg/mL)	$y = 4E{+}07x + 1E{+}06$	0.9909	0.29	0.96
Citric acid	0.04-5 (mg/mL)	$y = 1E{+}06x - 10277$	0.9997	4.4	14.5
Shikimic acid	0.04-5 (mg/mL)	$y = 5E{+}07x + 109778$	0.9999	10.2	56.5
Chlorogenic acid	2.5–80 (μg/mL)	$y = 312503x – 199432$	0.9999	0.2	0.68
Ferulic acid	2.5–80 (μg/mL)	$y = 633126x – 185462$	0.9990	1.85	5.61
Sinapic acid	2.5–80 (μg/mL)	$y = 197337x + 30036$	0.9997	1.98	6.01
Epicatechin	25-800 (μg/mL)	$y = 10314x + 147331$	0.9994	22.8	78.3
Naringerin	25-800 (μg/mL)	$y = 18433x + 78903$	0.9998	18.7	56.6
Quercetin-3-*O*-glucoside	25–800 (μg/mL)	$y = 34843x – 160173$	0.9998	0.21	0.71

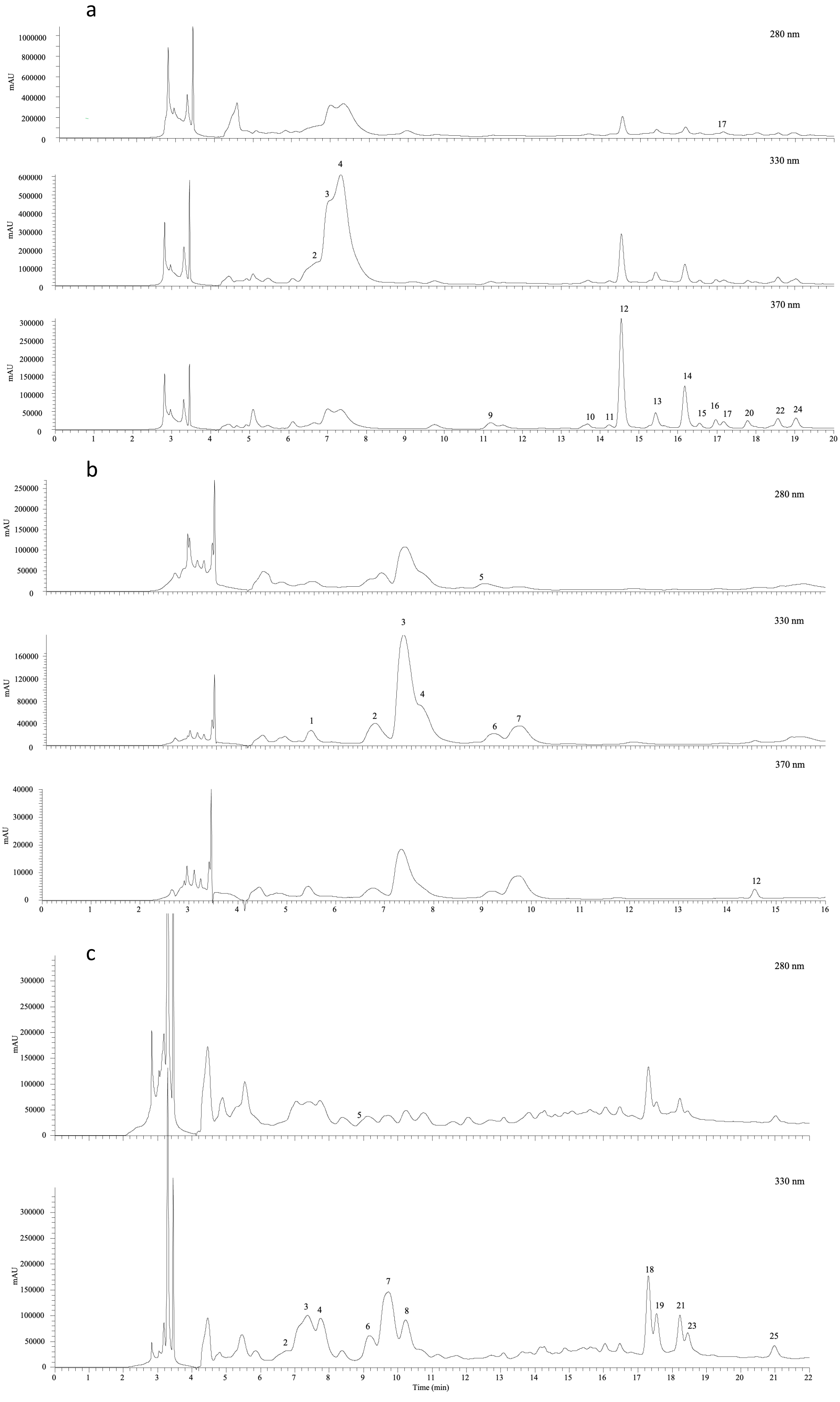

Figure S1. Chromatograms of *Solanum quitoense* peel (a), pulp (b), and seeds (c) hydroethanolic extracts recorded at 280, 330, and 370 nm.
